# Efficacy of ultrasound endoscopy with artificial intelligence for the differential diagnosis of non-gastric gastrointestinal stromal tumors

**DOI:** 10.1038/s41598-022-20863-8

**Published:** 2022-10-05

**Authors:** Yosuke Minoda, Eikichi Ihara, Nao Fujimori, Shuzaburo Nagatomo, Mitsuru Esaki, Yoshitaka Hata, Xiaopeng Bai, Yoshimasa Tanaka, Haruei Ogino, Takatoshi Chinen, Qingjiang Hu, Eiji Oki, Hidetaka Yamamoto, Yoshihiro Ogawa

**Affiliations:** 1 Department of Medicine and Bioregulatory Science, Graduate School of Medical Sciences, Kyushu University, 3-1-1 Maidashi, Higashi-Ku, Fukuoka, 812-8582 Japan; 2 Department of Endoscopic Diagnostics and Therapeutics, Kyushu University Hospital, 3-1-1 Maidashi, Higashi-Ku, Fukuoka, 812-8582 Japan; 3 Department of Gastroenterology and Metabolism, Graduate School of Medical Sciences, Kyushu University, 3-1-1 Maidashi, Higashi-Ku, Fukuoka, 812-8582 Japan; 4 Department of Surgery and Science, Graduate School of Medical Sciences, Kyushu University, 3-1-1 Maidashi, Higashi-Ku, Fukuoka, 812-8582 Japan; 5 Department of Pathological Sciences, Graduate School of Medical Sciences, Kyushu University, 3-1-1 Maidashi, Higashi-Ku, Fukuoka, 812-8582 Japan

**Keywords:** Cancer imaging, Gastrointestinal cancer

## Abstract

Gastrointestinal stromal tumors (GISTs) are common subepithelial lesions (SELs) and require treatment considering their malignant potential. We recently developed an endoscopic ultrasound-based artificial intelligence (EUS-AI) system to differentiate GISTs from non-GISTs in gastric SELs, which were used to train the system. We assessed whether the EUS-AI system designed for diagnosing gastric GISTs could be applied to non-gastric GISTs. Between January 2015 and January 2021, 52 patients with non-gastric SELs (esophagus, n = 15; duodenum, n = 26; colon, n = 11) were enrolled. The ability of EUS-AI to differentiate GISTs from non-GISTs in non-gastric SELs was examined. The accuracy, sensitivity, and specificity of EUS-AI for discriminating GISTs from non-GISTs in non-gastric SELs were 94.4%, 100%, and 86.1%, respectively, with an area under the curve of 0.98 based on the cutoff value set using the Youden index. In the subanalysis, the accuracy, sensitivity, and specificity of EUS-AI were highest in the esophagus (100%, 100%, 100%; duodenum, 96.2%, 100%, 0%; colon, 90.9%, 100%, 0%); the cutoff values were determined using the Youden index or the value determined using stomach cases. The diagnostic accuracy of EUS-AI increased as lesion size increased, regardless of lesion location. EUS-AI based on gastric SELs had good diagnostic ability for non-gastric GISTs.

## Introduction

Gastrointestinal stromal tumors (GISTs) are common subepithelial lesions (SELs) that are detected by screening endoscopy in approximately 0.4–3% of cases^1–3^. It is widely accepted that GISTs should be treated because of their malignant potential^4–6^. Although endoscopic ultrasound (EUS) has been reported as the most suitable modality for the diagnosis of SELs, it remains technically impossible to distinguish GISTs from non-GISTs using EUS alone^5,7^; thus, sampling of lesion tissue is required, such as via EUS-guided fine-needle aspiration/biopsy (EUS-FNAB)^4–6,8–11^.

Recently developed artificial intelligence (AI) techniques using deep learning methods, such as convolutional neural networks (CNNs), have been increasingly used in several medical fields. Accumulated evidence has shown that AI is a useful tool in clinical practice^12–15^. We recently reported the usefulness of an EUS-based AI system (EUS-AI) for distinguishing GISTs from non-GISTs in the stomach, where gastric SELs were used to train EUS-AI^16^. It is possible that EUS-AI will also be useful in the differential diagnosis of non-gastric GISTs if an EUS-AI system designed to evaluate each part of non-gastric SELs can be built. However, in order to build such EUS-AI systems, a large number of EUS SEL images, including GISTs, occurring in respective parts of the gastrointestinal tract are required. Indeed, substantial effort is required to collect non-gastric SEL images, including GISTs, since most SELs (and approximately 70% of gastrointestinal GISTs) occur in the stomach^17^. The first step is to assess whether EUS-AI designed for the diagnosis of gastric GISTs can be applied to non-gastric GISTs. Thus, the objective of the present study was to determine the diagnostic ability of EUS-AI that had been previously created based on gastric SELs for non-gastric SELs.

## Results

225 SEL cases was enrolled in this study. 52 patients with non-gastric SEL (15 esophageal, 26 duodenal, and 11 colon), including 20 patients diagnosed by EUS-FNAB or biopsy alone, were used as test cases (Table 1). The median size of the SELs enrolled in this study was 21.5 mm, and the efficacy of EUS-AI, which was constructed using 173 previously reported gastric SELs^16^, was assessed in two test cases: the abovementoned all 52 cases and 32 cases who underwent surgery with postoperative diagnosis of GIST in 31 cases and non-GIST in remaining only one excluding 20 cases being diagnosed by EUS-FNAB or biopsy alone.

**Table 1:** Patient and lesion characteristics for test cases.

Number of cases	52
Sex, male/female	33/19
Age, years	21.5 (32–74)
Lesion size, mm	21.5 (8.0–100)
**Histological type and number of cases**	
GIST	36 (93.3%)
Leiomyoma	14 (6.7%)
Aberrant pancreas	1 (3.8%)
Appendiceal mucocele	1 (9.1%)

Values are presented as n, n (%), or median (range).

GIST, gastrointestinal stromal tumors.

**Figure 1:**
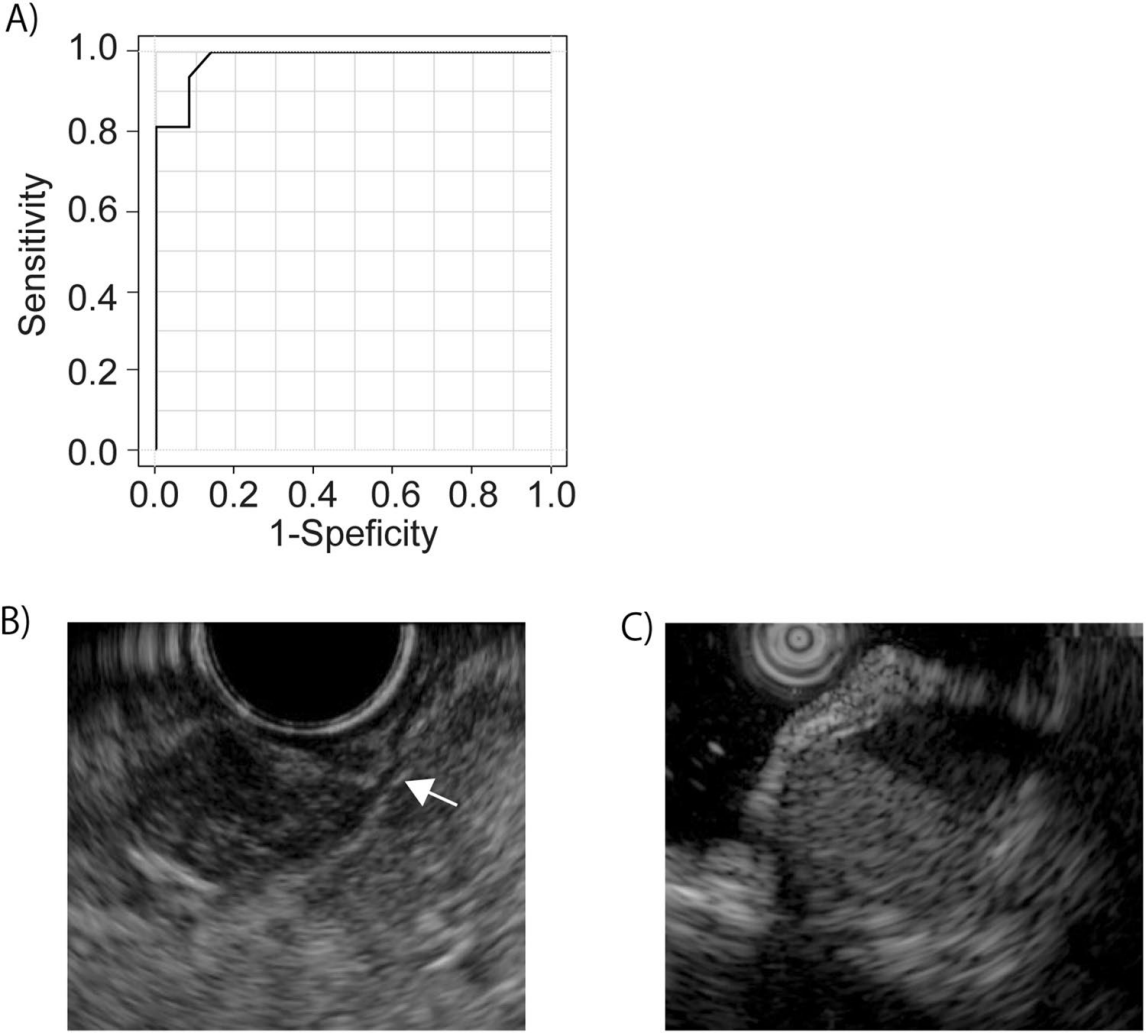
Diagnostic ability of EUS-AI. (**A**) The receiver operating characteristic curve for the diagnostic ability of EUS-AI for non-gastric GISTs is shown. Fifty-two patients with non-gastric SELs were analyzed using EUS-AI. For each case, a diagnosis of GIST or non-GIST was made, and the probability of GIST was evaluated. (**B**) EUS image of a duodenal aberrant pancreas, which was misdiagnosed as GIST by EUS-AI. The lesion was judged to originate from the muscle layer (white arrow). (**C**) EUS image of colonic appendiceal mucosal retention, which was misdiagnosed as GIST by EUS-AI. EUS-AI, endoscopic ultrasound-based artificial intelligence; GIST, gastrointestinal stromal tumors; SEL, subepithelial lesion.

When evaluated in all 52 cases, the accuracy, sensitivity, and specificity of EUS-AI for discriminating GISTs from non-GISTs in the gastrointestinal tract other than the stomach were 94.4%, 100%, and 86.1%, respectively. The receiver operating characteristic curve of EUS-AI for the diagnosis of non-gastric GISTs and its AUC (0.98, high accuracy) are shown in Fig. 1. When evaluated in 32 cases where the final pathology diagnosis was made with surgical specimens, the accuracy, sensitivity, and specificity of EUS-AI was 96.9%,100%, and 0%, respectively, and its AUC was 0.90 (high accuracy). The null specificity was obtained because only one of non-GIST cases underwent surgery in this study population. In contrast, the diagnostic accuracy of EUS-FNAB was 100% for the 29 patients who underwent surgery after preoperative diagnosis had been made by EUS-FNAB.

In the subanalysis, the diagnostic abilities of EUS-AI for non-gastric GISTs in the esophagus, duodenum, and colon is analyzed using the abovementoned all 52 cases and summarized in Table 2. The accuracy, sensitivity, and specificity of EUS-AI for distinguishing GISTs from non-GISTs were 100%, 100%, and 100%, respectively, in the esophagus, 96.2%, 100%, and 0%, respectively, in the duodenum, and 90.9%, 100%, and 0%, respectively, in the colon based on the cutoff value set for each part of the gastrointestinal tract using the Youden index (Table 3). There was no difference in diagnostic accuracy and sensitivity for non-gastric GISTs in the esophagus, duodenum, and colon between the abovementioned Youden index and the cutoff value of 0.94 set for the stomach as previously reported. The AUC for the diagnostic ability of EUS-AI for non-gastric GISTs including the esophagus, duodenum, and colon were 1.0 (high accuracy), 0.96 (high accuracy), and 0.80 (moderate accuracy), respectively (Table 3).

**Table 2:** Patient and lesion characteristics of esophageal, duodenal, and colonic lesions.

**Esophagus**	
Number of cases	15
Sex, male/female	11/4
Age, years	45.5 (32–68)
Lesion size, mm	20.0 (8.0–55)
Histological type and number of cases	
Leiomyoma	14 (93.3%)
GIST	1 (6.7%)
**Duodenum**	
Number of cases	26
Sex, male/female	12/14
Age, years	65 (35–81)
Lesion size, mm	21.0 (13–39)
Histological type and number of cases	
GIST	25 (96.2%)
Aberrant pancreas	1 (3.8%)
**Colon**	
Number of cases	11
Sex, male/female	10/1
Age, years	55 (33–74)
Lesion size, mm	35.0 (12–100)
Histological type and number of cases	
GIST	10 (90.9%)
Appendiceal mucosal retention	1 (9.1%)

Values are presented as n, median (range), or n (%).

GIST, gastrointestinal stromal tumors.

**Table 3:** Diagnostic ability of EUS-AI according to cutoff values.

SEL location	Cases used to set the cutoff value	Cutoff values	Accuracy (%)	Sensitivity (%)	Specificity (%)	AUC
Esophagus	Esophageal	0.997	100	100	100	1.0
	Gastric	0.94	100	100	100	
Duodenum	Duodenal	0.93	96.2	100	0	0.96
	Gastric	0.94	96.2	100	0	
Colon	Colonic	0.93	90.9	100	0	0.80
	Gastric	0.94	90.9	100	0	

AUC, area under the curve; EUS-AI, endoscopic ultrasound-based artificial intelligence; SEL, subepithelial lesion.

### SEL cases that were incorrectly diagnosed by EUS-AI

Three cases were incorrectly diagnosed by EUS-AI as follows: esophageal leiomyoma 15 mm in size with mixed hypoechoic and hypoechoic features on EUS, duodenal aberrant pancreas (Fig. 1B), and colonic appendiceal myxoma (Fig. 1C).

### Relationship between the diagnostic accuracy of EUS-AI for non-gastric GISTs and lesion size

**Figure 2:**
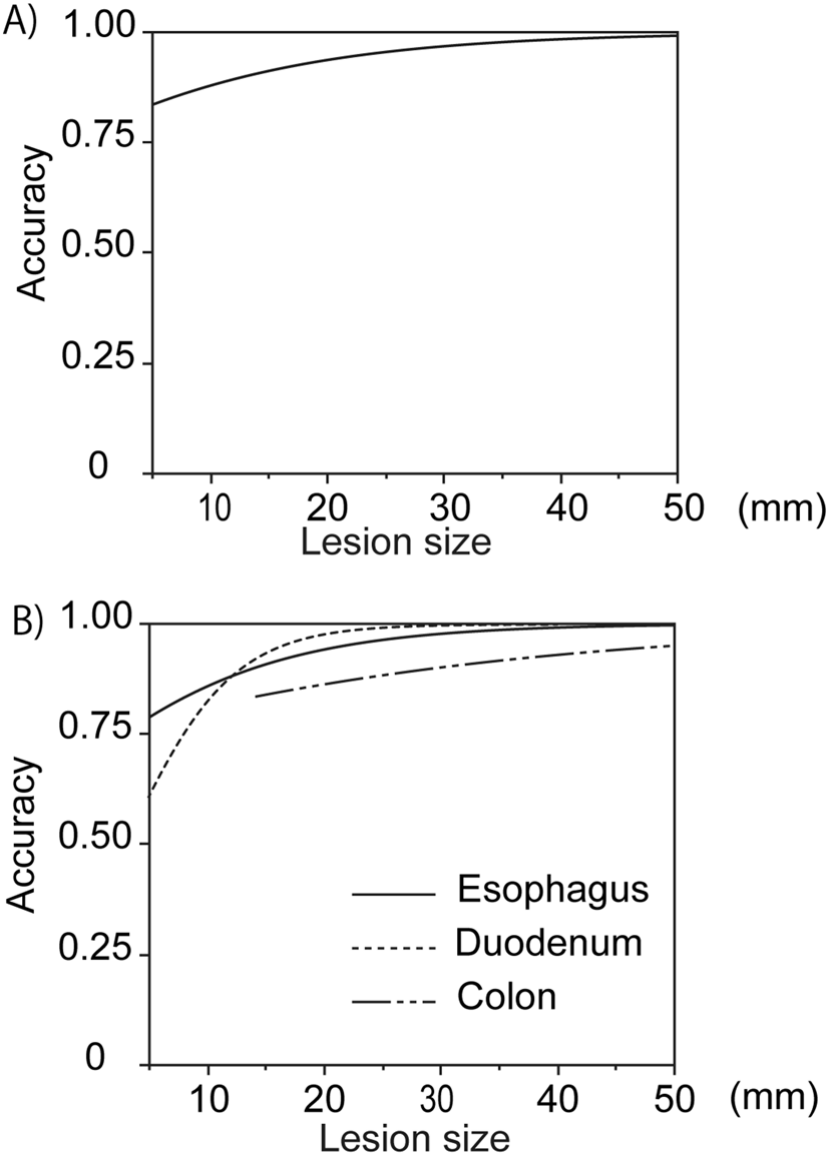
Relationship between the diagnostic ability of EUS-AI for non-gastric GISTs and lesion size. (**A**,**B**) The relationship between the diagnostic accuracy of EUS-AI and lesion size in the 52 cases with non-gastric SELs (**A**), and in each part of the gastrointestinal tract (esophagus; n = 15, duodenum; n = 26, colon; n = 11) (**B**) was examined using a logistic regression model. The concordance rates between the diagnoses of the abovementioned conditions and pathological results were determined. The cases with colorectal SELs had missing values between 0 and 12 mm because of the insufficient number of cases. EUS-AI, endoscopic ultrasound-based artificial intelligence; GIST, gastrointestinal stromal tumors; SEL, subepithelial lesion.

The relationship between the diagnostic accuracy of EUS-AI and lesion size is shown in Fig. 2A. The diagnostic accuracy of EUS-AI increased as the lesion size increased, regardless of the lesion location (Fig. 2B).

## Discussion

This study showed that EUS-AI is useful for the differential diagnosis of GISTs in the gastrointestinal tract other than the stomach, although it was trained for gastric SELs. Furthermore, the cutoff value of EUS-AI for distinguishing between GISTs and non-GISTs in the stomach can also be applied to non-gastric GISTs.

Even including the cases diagnosed by EUS-FNAB or biopsy alone, the diagnostic accuracy and sensitivity of EUS-AI for non-gastric GISTs were 90–100% and 100%, respectively, which are comparable to those of gastric GISTs (90% for accuracy and 100% for sensitivity) that we previously reported^16^, but also comparable to other AI systems with different algorithms for SELs of the upper gastrointestinal tract^18–20^. The diagnostic accuracy of EUS-AI for non-gastric GISTs in different parts of the gastrointestinal tract increased with lesion size, which was also consistent with the findings in the stomach, indicating that the diagnostic ability of EUS-AI depends on the lesion size regardless of tumor location. In addition, there was no significant difference in the diagnostic accuracy and sensitivity of EUS-AI for non-gastric GISTs between the cutoff value set for different part of the gastrointestinal tract using the Youden index and that of the stomach as we previously determined. These findings suggest that once the EUS-AI system is built to differentiate GISTs from non-GISTs in one part of the gastrointestinal tract, it can be applied to other parts of the gastrointestinal tract.

Interestingly, the diagnostic accuracy of EUS-AI was similar despite the large difference in the frequency of GISTs between the esophagus and other sites. This suggests that the frequency of the disease is not involved in EUS-AI diagnosis; instead, the EUS imaging findings themselves are important in diagnosis. EUS images of GISTs tend to show typical changes such as cystic degeneration with increasing lesion size^21–23^. It is possible that the accuracy of the EUS-AI system can be further improved by analyzing the pixel pattern of GISTs in the EUS image.

If the EUS-AI system constructed based on gastric SELs is established as the primary diagnostic tool not only for gastric GISTs but also for non-gastric GISTs, it will result in several advantages. First, EUS-AI is more feasible than EUS-FNAB for the diagnosis of duodenal SELs. Reports indicate that the diagnostic accuracy of EUS-FNAB for duodenal SELs is unsatisfactory owing to poor endoscope operability due to the unique duodenal anatomical structure of the narrow lumen and duodenal fold, although the malignant potential of duodenal GISTs is generally higher than that of gastric GISTs^24^. Indeed, it is much easier to obtain EUS images for EUS-AI than for EUS-FNAB. Second, EUS-AI can be safely applied without an invasive sampling process such as EUS-FNAB, and the differential diagnosis of GISTs can be automatically made, which is expected to reduce the risk of adverse events such as bleeding and infection. Third, EUS-AI can be used at any institution without the need for special endoscopic techniques, such as EUS-FNAB. This should lead to the early diagnosis and treatment of non-gastric GISTs, which are highly malignant^25^.

This study has some limitations. First, it was retrospective. Second, all EUS images were obtained from a single center. Third, the number of SELs was relatively small. Finally, we enrolled the case where the pathological diagnoses were made not only with surgical specimen but also with the samples obtained by EUS-FNAB or biopsy because the cases being diagnosed as benign tumors such as leiomyoma by EUS-FNAB or biopsy do not undergo surgery. It might cause a bias, however, we consider it is acceptable since the diagnostic accuracy of EUS-FNAB or biopsy in our institution could be sufficiently high. A prospective multicenter study should be required to verify the findings of the present study.

In conclusion, an EUS-AI system based on gastric SELs demonstrated good diagnostic ability for non-gastric GISTs. This system is likely to contribute to the diagnosis of gastrointestinal SELs in the future.

## Methods

### Study design and inclusion/exclusion criteria

This retrospective study was approved by the Kyushu University ethical review board on April 2021 (approval study number: 2021-190), and the requirement for obtaining written informed consent from patients was waived. All steps of this study were performed in accordance with relevant guidelines. A total of 225 SELs were included in this study, with the following inclusion criteria: patients with (a) non-gastric SELs who underwent EUS performed by EUS experts in our hospital between January 2015 and January 2021; (b) non-gastric SELs suspected to arise from the muscle layer of the gastrointestinal tract on EUS images, and (c) non-gastric SELs with available histopathological diagnoses. We excluded cases with poor-quality EUS images caused by air or strong artifacts.

### EUS images, lesion size, and pathological diagnosis

All EUS images were acquired by EUS experts with at least 5 years of experience in EUS procedures using conventional echoendoscopes (GF-UE260-AL5, GF-UCT260, TGF-UC260J; Olympus Corporation, Tokyo, Japan) or mini-probes (UM-3R, UM-2R; Olympus Corporation). All echoendoscopes and mini-probes were used while they were connected to conventional EUS processors (Prosound SSD α-75 scanner, Prosound SSD α-5 scanner: Hitachi Aloka, Tokyo, Japan; EU-ME2; Olympus Corporation). The maximum diameter of the lesions was measured using the EUS images. The histological diagnoses of the specimens obtained from EUS-FNAB, endoscopic biopsy, or surgical operation were made by three pathologists, including at least one certified by the Japanese Society of Pathology. No interobserver disagreement between the pathologists was reported for the diagnosis of SELs used in this study.

### AI training and construction of the EUS-AI system

The training method for the AI has been previously described^16^. Briefly, EUS images from 173 cases with gastric SELs were prepared to train the AI according to the following procedures. Each EUS image was trimmed to only the lesion to allow the AI to recognize the lesions. A total of 2718 images from 173 SELs in 173 cases (1729 images in 112 cases with GIST and 989 images of 61 non-GISTs, including 43 leiomyomas, 7 schwannomas, 7 ectopic pancreases, 2 lipomas, and 2 inflammatory changes) were prepared for the training data set.

Inverted forms and rotated images were prepared, with the images being rotated at 15° intervals; each rotated image was saved as a new image. The EUS-AI system was created on an AI-specific computer containing a GeForce GTX 1080 graphics processing unit (NVIDIA, California, USA), a core i7-8700 central processing unit (Intel, California, USA), and a deep analyzer graphical user interface-based deep learning tool (GHELIA, Tokyo, Japan). Xception was selected as the CNN-based deep learning model, and an adaptive moment estimation (ADAM) algorithm was selected to optimize the learning procedure.

### Diagnostic ability of EUS-AI for non-gastric GISTs

**Figure 3:**
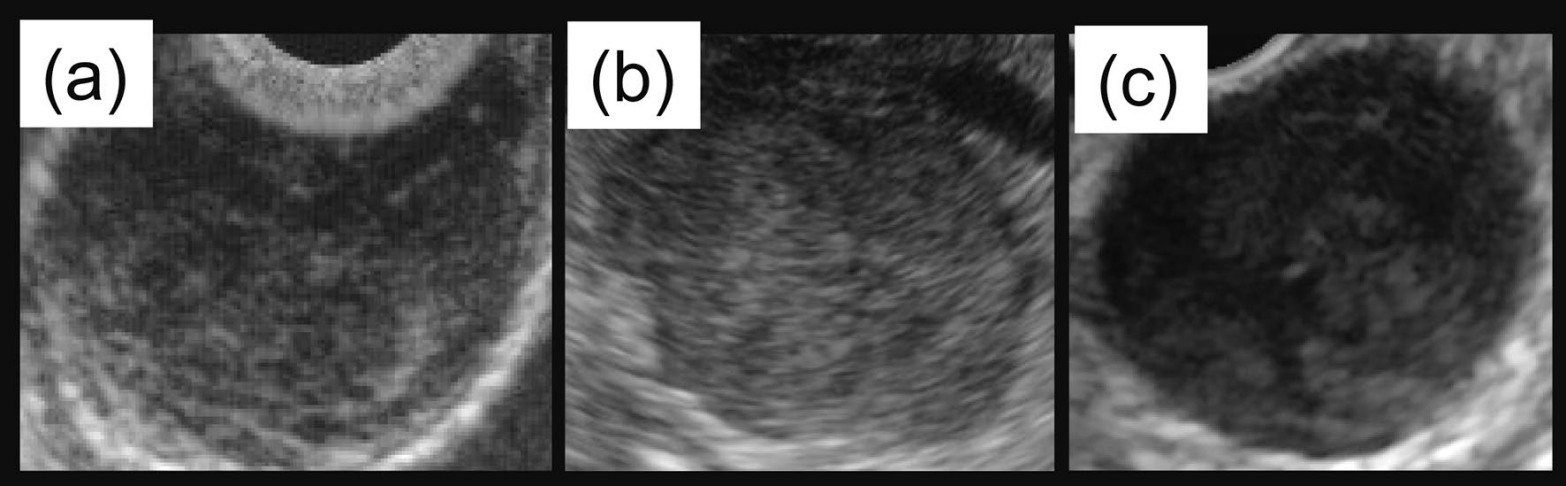
Representative EUS images of each lesion. Representative EUS images evaluated by EUS-AI were shown. (**a**) Esophageal leiomyoma, (**b**) duodenal GIST, (**c**) colonic GIST.

All EUS images for each case were supplied to the EUS-AI to make a diagnosis (Fig. 3). The EUS-AI estimated the probability that the lesion was a GIST on a scale of 0 to 1.0. The final diagnosis of GIST was made if the probability that the lesion was a GIST was higher than the cutoff value, which was set using the Youden index. The final diagnoses were assessed, and the diagnostic abilities of the EUS-AI system (accuracy, sensitivity, specificity, and area under the curve [AUC]) were evaluated. The diagnostic ability according to the AUC was defined as follows: 1.0–0.9 as high accuracy, 0.9–0.7 as moderate accuracy, and 0.7–0.5 as poor accuracy. All statistical analyses were performed using JMP software version 15.1 (SAS Institute, Cary, NC, USA). The diagnostic yield was also confirmed in cases where the postoperative pathological diagnosis was made in addition to determination of the diagnostic yield of EUS-AI using all non-gastric SEL cases. In addition, we evaluated the diagnostic accuracy of EUS-FNAB for the SEL cases who underwent surgery after preoperative diagnosis had been made by EUS-FNAB to determine that the pathological diagnosis made by EUS-FNAB is reliable in this study.

### Diagnostic ability of EUS-AI for non-gastric GISTs in parts of the gastrointestinal tract

To confirm whether there were any differences in the diagnostic ability of EUS-AI between the cutoff value set for each part of the gastrointestinal tract using the Youden index and that of the value set for the stomach (0.94) as we previously determined, the diagnostic abilities (accuracy, sensitivity, specificity, and AUC) calculated based on the two types of cutoff values were compared.

### Relationship between lesion size and diagnostic ability

To evaluate the relationship between the diagnostic ability of EUS-AI and lesion size, a subanalysis was conducted using the test sets, via a logistic regression model in the JMP software. In the logistic regression model, the independent variable was whether the diagnosis was correct, while the lesion size was set as the dependent variable.

## Data Availability

Of the datasets generated and analyzed in this study, data to the extent permitted by the Kyushu University Ethical Review Board are available from the corresponding authors upon reasonable request.
